# Socioeconomic status index is an independent determinant of breast cancer screening practices: Evidence from Eastern China

**DOI:** 10.1371/journal.pone.0279107

**Published:** 2022-12-14

**Authors:** Shunhua Zhang, Hairuo Wang, Binhao Liu, Jianwei Yu, Yizheng Gao

**Affiliations:** 1 School of Medical Imaging, Bengbu Medical College, Bengbu, Anhui, China; 2 School of Clinical Medicine, Bengbu Medical College, Bengbu, Anhui, China; Kaiser Permanente Washington, UNITED STATES

## Abstract

**Background:**

Breast cancer is the most prevalent malignancy affecting Chinese women, and early routine screening is incredibly important for its prevention and control. This study aimed to investigate the role of socioeconomic status (SES) in female breast cancer screening practices using the composite SES index.

**Methods:**

This cross-sectional study involved 1816 women in Eastern China. Data were collected using an online self-administered questionnaire from January 2020 to May 2021. We used principal component analysis to construct the composite SES index using educational level, annual household income, and occupation. Logistic regression was used to analyze the association between the SES index and breast cancer screening utilization.

**Results:**

Of the participants, 19.27% reported having performed breast self-examination, 12.89% reported having undergone clinical breast examination, and 3.52% reported having received mammography. The SES has a significant influence on the patronage of female breast cancer screening in Eastern China. The composite SES index was found to have a positive and significant association with breast cancer screening practices. An interaction was found between the SES index and patient characteristics, such as health awareness, marital status, and location of residence. In addition, the SES index had a positive effect on breast cancer-related knowledge.

**Conclusions:**

Socioeconomic inequalities existed in Eastern China and were related to breast cancer screening patronage. The composite SES index was identified as a significant determinant of breast cancer screening practices. Our results highlighted the negative impacts of socioeconomic inequities on female breast cancer screening utilization. This suggests that reducing socioeconomic inequalities in breast cancer screening requires more focused interventions and concerted outreach activities for groups with lower education levels, lower income, or lower occupational classes.

## Introduction

Female breast cancer ranks as the first commonly occurring malignancy in China [1, 2]. It poses a significant health threat to Chinese women. In 2020, 420,000 newly developed breast cancer cases were diagnosed in China, and 120,000 deaths were registered, accounting for 19.9% and 9.9% of the global female cancer incidence and mortality rate, respectively [1]. Because of the sustained socioeconomic development and rapid urbanization, China’s contribution to the global breast cancer rates is increasing every year, especially in younger women [3]. Within the past two decades, breast cancer incidence among Chinese women has increased by 3–5% annually, which is significantly higher than the average annual global increase of 0.5% [4]. The age-standardized rate of breast cancer in China increased from 21 to 29 per 100,000 women between 2003 and 2011 [5], with large differences between urban and rural regions [6, 7]. Obviously, breast cancer prevention and control are challenging in China.

Early routine screening is considered imperative to reduce the mortality rate of this highly treatable malignant disease. The breast cancer mortality rate in the United States decreased by 40% between 1989 and 2017, mainly due to increased screening utilization [8]. Furthermore, a study by McCormack et al. [9] in 5 sub-Saharan African countries reported that an estimated 28–37% of breast cancer-related deaths can be prevented through early detection and adequate treatment. Therefore, close attention must be paid to the screening behaviors of women for early prevention and treatment of breast cancers.

Screening for early detection of breast cancer has been widely promoted elsewhere [10–12], but it has not been prioritized in China. Currently, there has been no organized national breast-screening program; the Chinese government does not offer free screening services for women [13]. Additionally, breast cancer screenings are not covered by the Chinese universal healthcare insurance. The China Anti-Cancer Association (CACA) has encouraged asymptomatic women older than 20 years to carry out BSE monthly [14]. Simultaneously, women older than 40 years, or younger if they have a high risk, are recommended to undergo mammography in combination with CBE or ultrasonography every 1 to 2 years [14]. However, breast cancer screening rates have remained low among Chinese women [15–18]. A survey conducted in 2010 reported that only 21.7% of 53,513 Chinese women aged 18–107 years had been screened [17]. A 2013 large-scale national study found that only 22.5% of Chinese women aged 35–69 years were screened [18]. Therefore, gaining a better understanding of the determinants of breast cancer screening is important to improve screening rates.

Evidence from different countries and territories (including Qatar and Latin America) suggests that low socioeconomic status (SES) is a substantial barrier to the utilization of breast cancer screening [19–23]. Women with lower SES have access to a smaller pool of health promotion resources [24], which may limit their participation in health-promoting programs such as breast cancer screening. Furthermore, a study in China reported that patients with lower SES had a later breast cancer stage at the time of diagnosis and a poor overall prognosis [25]. Several socioeconomic factors, including lower income, education and occupation, residence in rural areas, and single marital status, have been determined to be strong predictors of low patronage of breast cancer screening services [19–23]. However, research on the influence of SES on breast cancer screening patronage among women in China is limited.

SES is a robust determinant of health promotion programs, referring to the position of an individual in the social stratification system [26]. SES is generally measured using educational attainment, income, occupation, or a composite of these variables [27]. Using single SES variables can only improve the understanding of the relationship between a certain aspect of SES and breast cancer screening. However, SES is inherently complex and multidimensional [27]. Individual SES variables cannot capture the entire concept of SES [28]. The SES index is a composite measure, which can overcome the aforementioned problems [27]. It is generally constructed using a combination of SES variables [27]. Nevertheless, few studies have investigated the association between the composite SES and breast cancer screening patronage among Chinese women. It is also not clear whether the SES index is a stable variable and whether it changes the association of SES indicators with breast cancer screenings.

This study aimed to assess the association between the SES index and female breast cancer screening practices in Eastern China, while considering the differences in screening utilization of women with different SES. We hypothesized that the SES index may significantly affect female breast cancer screening patronage, and women with high SES may be more likely to engage in breast cancer screenings.

## Materials and methods

### Design and participants

We carried out this cross-sectional survey in Anhui, Eastern China. A snowball sampling strategy was utilized.

Data on breast cancer screening practices questions for most countries were limited to women of reproductive age (15–50 years old) [17, 22, 23, 29–33]. In addition, given the increasing incidence of breast cancer and considering the onset age for breast cancer at younger age in Chinese women [3–7], we conducted this study targeting Chinese women aged 18 to 70.

We distributed an anonymous web-based questionnaire through WeChat/Weixin (version 8.0.11) between January 2020 and May 2021. We encouraged the respondents who had received the link to the survey to forward it to their friends and relatives. Before the survey, all participants read the study objectives and consent information and provided implied consent after clicking “Continue” to complete the online questionnaire. On average, it took 10–15 minutes for participants to complete the survey. Participants who completed the survey were rewarded with electronic cashable coupons. Participants who were registered but did not live in Anhui and had a history of breast cancer and cognitive impairment were excluded. Duplicated responses were blocked by checking the unique ID of each participant. De-identified data were collected and analyzed to ensure participant confidentiality.

We computed the sample size using a 95% confidence interval and a statistical formula [34]. Considering a possible invalid response rate (20%), a minimum sample size of 461 was determined. A total of 2,223 eligible participants were voluntarily enrolled in our study. Data from the 407 questionnaires were excluded due to missing values (>10%). Ultimately, we obtained 1,816 complete and valid questionnaires for further analyses, yielding an effective response rate of 81.69%. The sample size of this study exceeded the minimum requirement. A sufficient sample size ensures the credibility and repeatability of our results.

The Ethics Committee of Bengbu Medical College reviewed and approved the protocol of this study (No. 2021–070). The study was conducted in accordance with the ethical standards of the institutional and national research committee and the Declaration of Helsinki.

### Measurements

The initial questionnaire was developed based on our previous studies [16, 35], and an extensive literature review [19–23]. We assessed content validity by sending the first draft of the questionnaire to two public health experts and two breast cancer experts. Each expert was invited to rate each item based on the relevance of the item content. They were also asked to give comments on the general formulations of the initial questionnaire. We conducted a small-scale pilot survey from a convenience sample of 90 women of different ages. Those who gave informed consent voluntarily and met the inclusion criteria were included. They were required to complete the questionnaire, to comment regarding the ease of understanding and acceptability of each item. We improved the questionnaire according to the expert’s recommendations and the pre-survey results. The actual study samples did not include the pilot population.

We conducted the exploratory factor analysis (EFA) and reliability test to assess construct validity and internal consistency reliability. The Kaiser-Meyer-Olkin (KMO) value of the questionnaire was 0.876, and bartlett’s test of sphericity value for *p* was 0.000. EFA with iterated principal factor extraction resulted in 4 factors with initial eigenvalue estimates above 1.0 and 64.229% variance explained. The Cronbach’s alpha coefficients was 0.780.

A 2‑week test–retest examination was conducted with the participation of 50 volunteers from a pilot sample. During this time, participants could not consult the replies they had given when the questionnaire was first administered. We used the Cohen’s Kappa to assess test-retest reliability, and the Cohen’s kappa value was 0.53–0.79.

The final questionnaire included items for the sociodemographic profile, SES, breast cancer knowledge, and breast cancer screening practices.

Sociodemographic data, including age, marital status, number of children, location of residence, education level, annual household income, and occupation, were collected. Participants were also asked whether they had received breast health education.

SES was assessed using three direct indicators, which were education level, annual household income, and occupation, and one composite variable, which was the SES index. Five education level categories were used: junior high school or below, high school or vocational school, junior college, university, and masters and above [36]. We used three income levels to assess annual household income: <50,000 CNY (Chinese yuan renminbi), 50,000–120,000 CNY, and >120,000 CNY. Occupation was classified into two major groups: non-high-tech (the unemployed, manual workers, farmers, service providers, and ordinary staff) and high-tech (senior professional and technical personnel, leaders of government agencies, and senior executives) [37, 38]. We conducted a factor analysis to construct the composite SES index using the three SES indicators [39]. We utilized a varimax rotation with Kaiser normalization. The principal component analysis was used as the extraction method [39–41]. The principal component analysis results showed that the Kaiser-Meyer-Olkin (KMO) value was 0.661, and Bartlett’s sphere test was significant (*p* < 0.001). This indicated a correlation among three SES indicators. Eigenvalues of >1 were applied as the extraction criteria [41]. The results showed that only the first principal component was extracted, which explained 61.34% of the total variance variation. Each indicator score was multiplied by its standardized factor loading. Referring to previous studies [39–41], we calculated a composite SES index (SESI = Zoccupation * 0.417 + Zeducation * 0.442 + Zincome * 0.418) / 0.613. We applied the median to categorize the SES index scores into low, medium, and high.

We used ten questions to assess breast cancer knowledge, covering breast cancer risk factors, possible symptoms, and screening strategies. The responses had to be “true” or “false”. A score of 1 was assigned for the correct response, while incorrect responses were scored 0. Higher total knowledge scores (range, 0–10) represented higher levels of breast cancer knowledge.

We used three questions to ask participants about their breast cancer screening experiences. The questions included the following: “Have you performed BSE regularly during the past 12 months?”; “Have you undergone CBE in the past 2 years?”; and “Have you received mammography screening within the past 2 years?”. The answers had to be “Yes” or “No”. BSE was defined as ‘‘a woman examines her breasts”. CBE was defined as “a health professional uses his/her hands to detect breast changes”. Mammography was defined as “a radiographic imaging of the breast by a mammography machine”.

### Statistical analysis

We used IBM® SPSS® Statistics (version 22.0, IBM Corporation, Chicago, IL, USA) for data analysis. We applied *χ^2^* tests to observe the proportions of breast cancer screening programs by different types of personnel. We used the Kruskal-Wallis test to examine the differences in breast cancer knowledge because the data did not follow a normal distribution. We used a bivariate Spearman’s correlation analysis to evaluate the potential correlations between socioeconomic factors and breast cancer screenings. We utilized multivariate logistic regression to estimate the association between the SES index and female breast cancer screening utilization, applying the forward stepwise method. Three multivariate regression analyses were performed to determine whether breast health education, marital status, and location of residence significantly affected breast cancer screening patronage after accounting for the SES index. The same order of entry was utilized for three sets of regression analyses, with the SES index entered in step 1, followed by the breast health education in step 2, and lastly, marital status and location of residence entered in step 3. Before introducing independent variables into the regression analysis, the variables were assessed for multicollinearity and interactions.

Conservatively, the two-tailed *p*-value of <0.05 was considered statistically significant.

## Results

### Demographic information of participants

**Fig 1:**
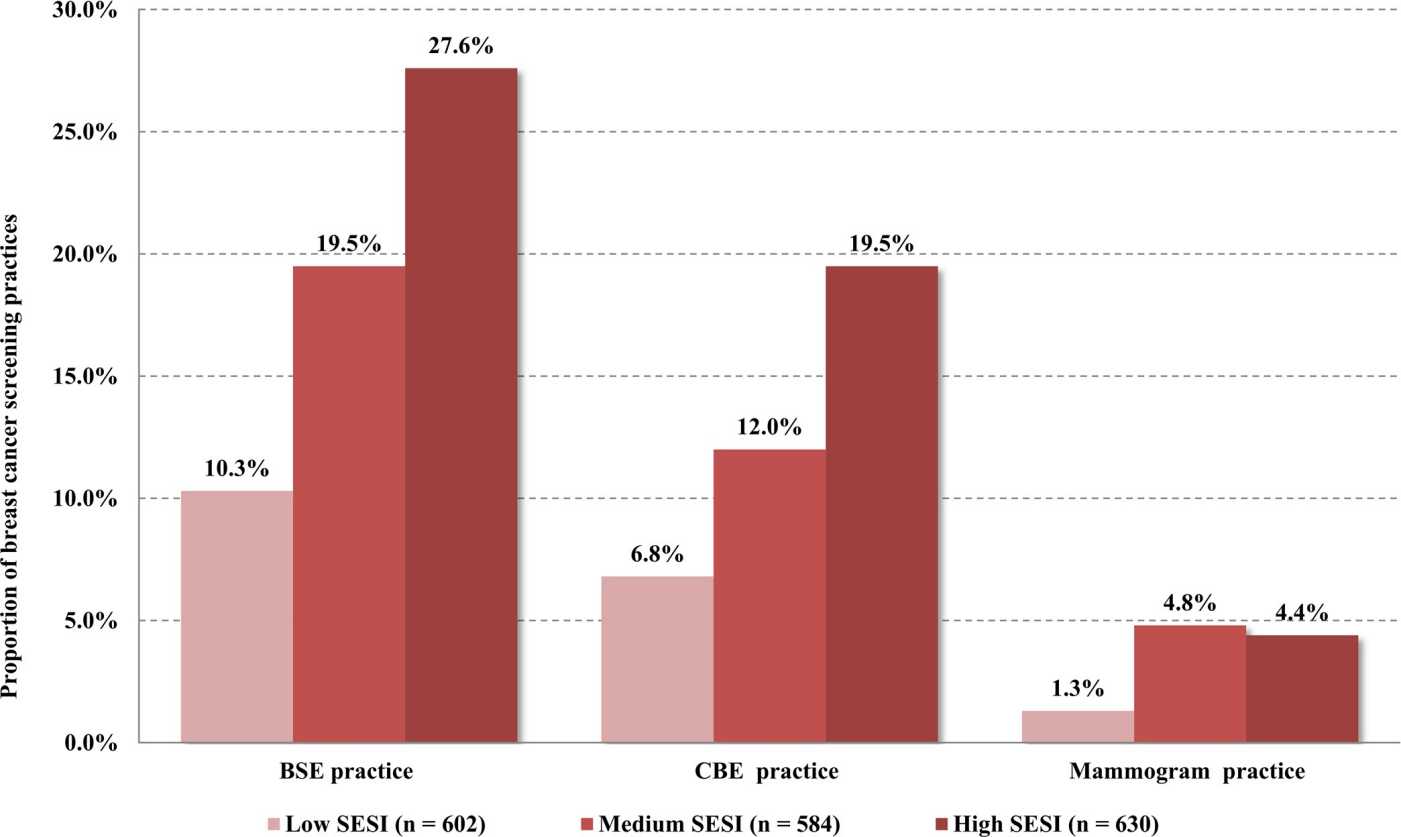
Proportion of breast cancer screening practices (n = 1816). BSE, breast self-examination; CBE, clinical breast examination; SESI, socioeconomic status index.

The average age of 1,816 participants was 34.01 years (standard deviation, SD = 10.97) and ranged from 19 to 70 years in this study. Of the total sample, 41.2% were university–educated, 86.3% had an annual household income of 50,000 or higher, 52.9% worked in high-tech jobs, 19.27% (n = 350) reported having practiced BSE, 12.89% (n = 234) indicated ever having undergone CBE, and 3.52% (n = 64) reported having received mammography. The rate of BSE was higher than those of CBE and mammography. In comparison, married urban women who had children received breast health education showed higher rates of breast cancer screening patronage. The results of the *χ^2^* tests showed that breast cancer screening patronage was significantly correlated with most of the sociodemographic variables. Except for mammography, the rates of BSE and CBE increased with the improvement of the SES index (all *p*<0.001). Fig 1 shows the proportions of BSE, CBE, and mammography among women with different SES indexes. Another sociodemographic profile is reported in Table 1.

**Table 1:** Demographic information and breast cancer screening practices (n = 1816).

Variables	Total (%)	BSE practice (%)		*p*-Value	CBE practice (%)		*p*-Value	Mammography practice (%)		*p*-Value
		Yes (n = 350)	No (n = 1466)		Yes (n = 234)	No (n = 1582)		Yes (n = 64)	No (n = 1752)	
**Age (years)**				0.000 ***			0.000 ***			0.373
18–29	826 (45.5)	112 (13.6)	714 (86.4)		74 (9.0)	752 (91.0)		25 (3.0)	801 (97.0)	
30–39	432 (23.8)	80 (18.5)	352 (81.5)		57 (13.2)	375 (86.8)		13 (3.0)	419 (97.0)	
40–49	334 (18.4)	97 (29.0)	237 (71.0)		65 (19.5)	269 (80.5)		15 (4.5)	319 (95.5)	
≥50	224 (12.3)	61 (27.2)	163 (72.8)		38 (17.0)	186 (83.0)		11 (4.9)	213 (95.1)	
**Marital status**				0.000 ***			0.000 ***			0.307
Single	707 (38.9)	86 (12.2)	621 (87.8)		61 (8.6)	646 (91.4)		21 (3.0)	686 (97.0)	
Married	1109 (61.1)	264 (23.8)	845 (76.2)		173 (15.6)	936 (84.4)		43 (3.9)	1066 (96.1)	
**Number of children**				0.000 ***			0.000 ***			0.544
0 (none)	805 (44.3)	106 (13.2)	699 (86.8)		78 (9.7)	727 (90.3)		26 (3.2)	779 (96.8)	
≥ 1 child	1011 (55.7)	244 (24.1)	767 (75.9)		156 (15.4)	855 (84.6)		38 (3.8)	973 (96.2)	
**Location of residence**				0.000 ***			0.000 ***			0.001 **
Rural areas	828 (45.6)	101 (12.2)	727 (87.8)		62 (7.5)	766 (92.5)		16 (1.9)	812 (98.1)	
Urban areas	988 (54.4)	249 (25.2)	739 (74.8)		172 (17.4)	816 (82.6)		48 (4.9)	940 (95.1)	
**Ever received breast health education**				0.000 ***			0.000 ***			0.002 **
No	969 (53.4)	104 (10.7)	865 (89.3)		83 (8.6)	886 (91.4)		22 (2.3)	947 (97.7)	
Yes	847 (46.6)	246 (29.0)	601 (71.0)		151 (17.8)	696 (82.2)		42 (5.0)	805 (95.0)	
**Education level**				0.000 ***			0.000 ***			0.019 *
Junior middle school and below	199 (11.0)	7 (3.5)	192 (96.5)		7 (3.5)	192 (96.5)		2 (1.0)	197 (99.9)	
Senior middle school and technical school	168 (9.3)	30 (17.9)	138 (82.1)		18 (10.7)	150 (89.3)		1 (0.6)	167 (99.4)	
Junior college	700 (38.5)	123 (17.6)	577 (82.4)		73 (10.4)	627 (89.6)		28 (4.0)	672 (96.0)	
University	660 (36.3)	164 (24.8)	496 (75.2)		114 (17.3)	546 (82.7)		27 (4.1)	633 (95.9)	
Master and above	89 (4.9)	26 (29.2)	63 (70.8)		22 (24.7)	67 (75.3)		6 (6.7)	83 (93.3)	
**Annual household income**				0.000 ***			0.000 ***			0.000 ***
< 50000 CNY	249 (13.7)	20 (8.0)	229 (92.0)		18 (7.2)	231 (92.8)		5 (2.0)	244 (98.0)	
50000 CNY-120000 CNY	1033 (56.9)	178 (17.2)	855 (82.8)		116 (11.2)	917 (88.8)		27 (2.6)	1006 (97.4)	
>120000 CNY	534 (29.4)	152 (28.5)	382 (71.5)		100 (18.7)	434 (81.3)		32 (6.0)	502 (94.0)	
**Occupation**				0.000 ***			0.000 ***			0.002 **
Non-high-tech	856 (47.1)	94 (11.0)	762 (89.0)		67 (7.8)	789 (92.2)		18 (2.1)	838 (97.9)	
High-tech	960 (52.9)	256 (26.7)	704 (73.3)		167 (17.4)	793 (82.6)		46 (4.8)	914 (95.2)	
**SESI**				0.000 ***			0.000 ***			0.001 **
Low	602 (33.1)	62 (10.3)	540 (89.7)		41 (6.8)	561 (93.2)		8 (1.3)	594 (98.7)	
Medium	584 (32.2)	114 (19.5)	470 (80.5)		70 (12.0)	514 (88.0)		28 (4.8)	556 (95.2)	
High	630 (34.7)	174 (27.6)	456 (72.4)		123 (19.5)	507 (80.5)		28 (4.4)	602 (95.6)	

BSE, breast self-examination; CBE, clinical breast examination; CNY, Chinese yuan renminbi; SESI, socioeconomic status index.

Data are expressed as n (%).

*P*-Values were calculated using Pearson’s *χ^2^* test. *** *p* < 0.001, ** *p* < 0.01, * *p* < 0.05.

### SES index and breast cancer knowledge

**Fig 2:**
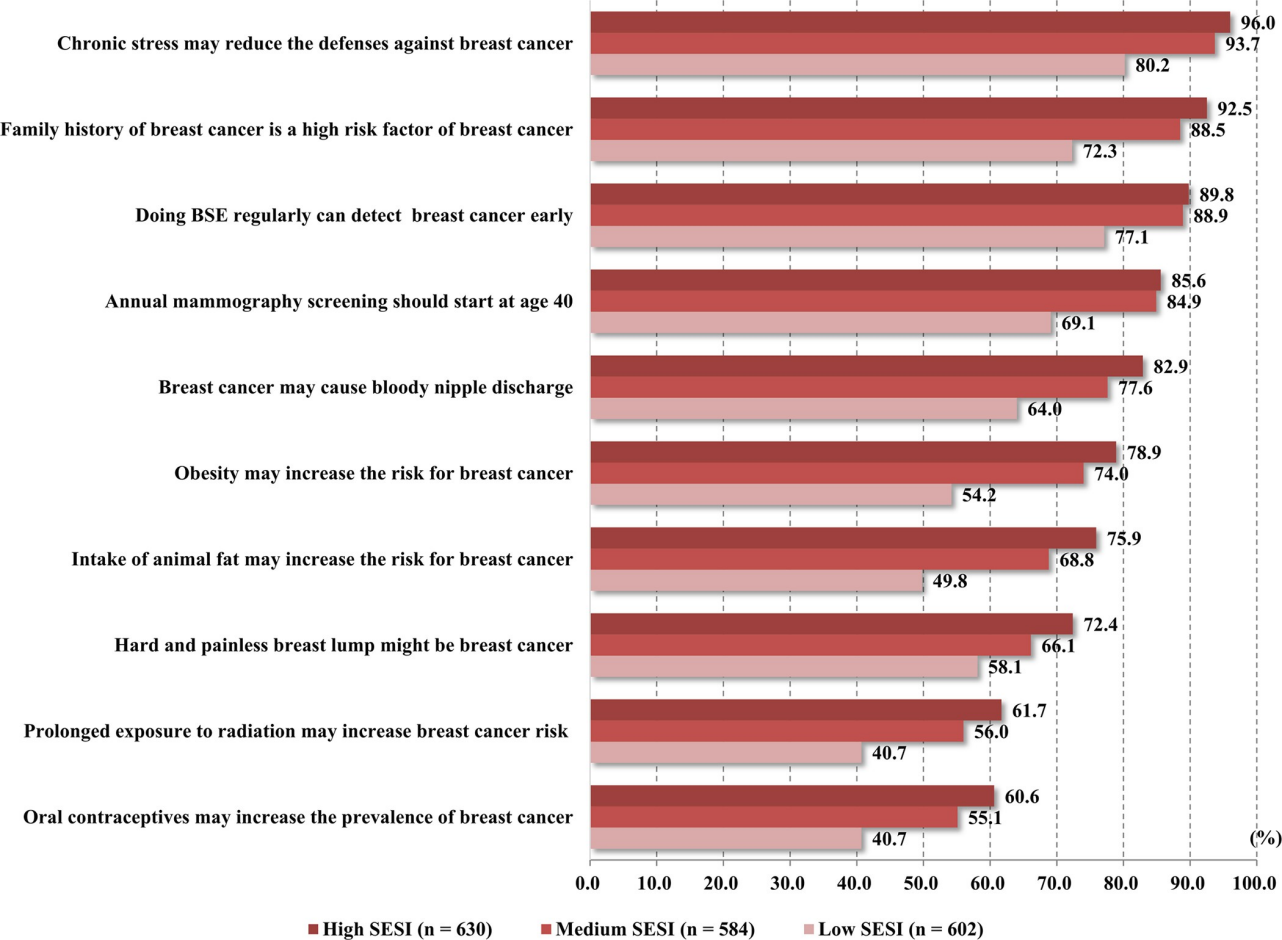
Proportion of correct responses for breast cancer knowledge (n = 1816). SESI, socioeconomic status index.

Fig 2 depicts the proportion of correct responses about breast cancer knowledge among women with different SES indexes. The proportion of correct responses to all knowledge questions was higher for women with higher SES indexes than those with lower SES indexes (all *p* < 0.001). Furthermore, results from the Kruskal-Wallis test showed that the mean total knowledge score of women with high SES indexes was 7.96 (SD ± 1.99), and it was significantly higher *(χ^2^* = 209.819, *p* < 0.001) than the score of women with medium (7.54 ± 2.06) and low (6.06 ± 2.61) SES indexes. The comparison of breast cancer knowledge among women with different SES indexes is provided in Table 2.

**Table 2:** Comparison of breast cancer knowledge among women with different SESI (n = 1816).

Questions	High SESI (*n* = 630)	Medium SESI (*n* = 584)	Low SESI (*n* = 602)	Chi-square (*χ*^*2*^)	p-Value
Total knowledge score, mean (SD)	7.96 (2.0)	7.54 (2.1)	6.06 (2.6)	209.819	0.000 ^a^
Chronic stress may reduce the defenses against breast cancer (%)	605 (96.0)	547 (93.7)	483 (80.2)	98.281	0.000 ^b^
Family history of breast cancer is a high risk factor of breast cancer (%)	583 (92.5)	517 (88.5)	435 (72.3)	107.342	0.000 ^b^
Doing BSE regularly can detect breast cancer early (%)	566 (89.8)	519 (88.9)	464 (77.1)	48.758	0.000 ^b^
Annual mammography screening should start at age 40 (%)	539 (85.6)	496 (84.9)	416 (69.1)	65.453	0.000 ^b^
Breast cancer may cause bloody nipple discharge (%)	522 (82.9)	453 (77.6)	385 (64.0)	61.783	0.000 ^b^
Obesity may increase the risk for breast cancer (%)	497 (78.9)	432 (74.0)	326 (54.2)	97.772	0.000 ^b^
Intake of animal fat may increase the risk for breast cancer (%)	478 (75.9)	402 (68.8)	300 (49.8)	97.351	0.000 ^b^
Hard and painless breast lump might be breast cancer (%)	456 (72.4)	386 (66.1)	350 (58.1)	27.762	0.000 ^b^
Prolonged exposure to radiation may increase breast cancer risk (%)	389 (61.7)	327 (56.0)	245 (40.7)	58.006	0.000 ^b^
Oral contraceptives may increase the prevalence of breast cancer (%)	382 (60.6)	322 (55.1)	245 (40.7)	51.906	0.000 ^b^

SESI, socioeconomic status index; BSE, breast self-examination.

^a^ Kruskal-Wallis test

^b^ Chi square test.

Data are expressed as mean ± SD, and n (%). SD, standard deviation.

### Bivariate correlation analysis of socioeconomic factors and breast cancer screening

Spearman’s correlation analysis indicated that education level, income, occupation, and the composite SES index were all positively associated with the three breast cancer screening practices (all *p* < 0.01). Table 3 shows the specific Spearman correlation coefficients.

**Table 3:** Bivariate correlations between socioeconomic factors and breast cancer screening practices (n = 1816).

Variables	BSE practice	CBE practice	Mammography practice
Education level	0.154 **	0.146 **	0.065 **
Annual household income	0.169 **	0.118 **	0.087 **
Occupation	0.199 **	0.143 **	0.073 **
SESI	0.201 **	0.174 **	0.077 **

BSE, breast self-examination; CBE, clinical breast examination; SESI, socioeconomic status index.

** *p* < 0.01. *P*-Values were calculated using Spearman’s correlation analysis.

### Associations of the SES index with breast cancer screening practices

Tables 4–6 summarizes the associations of the SES index with the three breast cancer screening practices. The results indicated that the SES index was statistically significantly positively associated with female breast cancer screening practices.

**Table 4:** Multivariate logistic regressions of BSE practice by SES index (n = 1816).

Variables	Model 1		Model 2		Model 3	
	OR (95% CI) ^a^	*p*-Value	OR (95% CI) ^b^	*p*-Value	OR (95% CI) ^c^	*p*-Value
**Step 1**						
**SESI**						
Low	1.000		1.000		1.000	
Medium	2.113 (1.514–2.948)	0.000 ***	1.674 (1.189–2.358)	0.003 **	1.968 (1.375–2.817)	0.000 ***
High	3.323 (2.424–4.556)	0.000 ***	2.674 (1.934–3.698)	0.000 ***	2.376 (1.695–3.330)	0.000 ***
**Step 2**						
**Ever received breast health education**						
No			1.000		1.000	
Yes			3.017 (2.333–3.902)	0.000	2.903 (2.236–3.770)	0.000 ***
**Step 3**						
**Marital status**						
Single					1.000	
Married					2.183 (1.630–2.923)	0.000 ***
**Location of residence**						
Rural areas					1.000	
Urban areas					1.484 (1.122–1.963)	0.006 **

BSE, breast self-examination; SESI, socioeconomic status index; OR, odds ratio.

^a^ Crude OR

^b^ Adjusted for health education

^c^ Adjusted for breast health education, marital status, and location of residence.

Step 1 model fit, −2 log likelihood = 1718.415, Cox and Snell R^2^ = 0.033, Nagelkerke R^2^ = 0.054, Model *χ*^*2*^ *=* 61.843 (*p* < 0.001).

Step 2 model fit, −2 Log likelihood = 1642.570, Cox and Snell R^2^ = 0.073, Nagelkerke R^2^ = 0.117, Model *χ*^*2*^ *=* 137.689 (*p* < 0.001).

Step 3 model fit, −2 log likelihood = 1594.133, Cox and Snell R^2^ = 0.097, Nagelkerke R^2^ = 0.156, Model *χ*^*2*^ *=* 186.125 (*p* < 0.001).

*P*-Values were calculated using logistic regression analysis.

*** *p* < 0.001

** *p* < 0.01.

**Table 5:** Multivariate logistic regressions of CBE practice by SES index (n = 1816).

Variables	Model 1		Model 2		Model 3	
	OR (95% CI) ^a^	*p*-Value	OR (95% CI) ^b^	*p*-Value	OR (95% CI) ^c^	*p*-Value
**Step 1**						
**SESI**						
Low	1.000		1.000		1.000	
Medium	1.863 (1.245–2.790)	0.003 **	1.591 (1.055–2.398)	0.027 *	1.705 (1.114–2.611)	0.014 *
High	3.320 (2.285–4.822)	0.000 ***	2.845 (1.946–4.160)	0.000 ***	2.410 (1.626–3.573)	0.000 ***
**Step 2**						
**Ever received breast health education**						
No			1.000		1.000	
Yes			2.029 (1.514–2.718)	0.000 ***	1.909 (1.421–2.566)	0.000 ***
**Step 3**						
**Marital status**						
Single					1.000	
Married					1.693 (1.212–2.364)	0.002 **
**Location of residence**						
Rural areas					1.000	
Urban areas					1.725 (1.238–2.404)	0.001**

CBE, clinical breast examination; SESI, socioeconomic status index; OR, odds ratio.

^a^ Crude OR

^b^ Adjusted for health education

^c^ Adjusted for breast health education, marital status, and location of residence.

Step 1 model fit, −2 log likelihood = 1349.799, Cox and Snell R^2^ = 0.025, Nagelkerke R^2^ = 0.046, Model *χ*^*2*^ = 45.628 (*p* < 0.001).

Step 2 model fit, −2 Log likelihood = 1326.520, Cox and Snell R^2 =^ 0.037; Nagelkerke R^2^ = 0.069, Model *χ*^*2*^ = 68.907 (*p* < 0.001).

Step 3 model fit, −2 log likelihood = 1298.249, Cox and Snell R^2^ = 0.052; Nagelkerke R^2^ = 0.097, Model *χ*^*2*^ = 97.178 (*p* < 0.001).

*P*-Values were calculated using logistic regression analysis.

*** *p* < 0.001

** *p* < 0.01

* *p* < 0.05.

**Table 6:** Multivariate logistic regressions of mammography practice by SES index (n = 1816).

Variables	Model 1		Model 2		Model 3	
	OR (95% CI) ^a^	*p*-Value	OR (95% CI) ^b^	*p*-Value	OR (95% CI) ^c^	*p*-Value
**Step 1**						
**SESI**						
Low	1.000		1.000		1.000	
Medium	3.739 (1.690–8.274)	0.001 **	3.242 (1.452–7.241)	0.004 **	3.179 (1.395–7.243)	0.006 **
High	3.453 (1.561–7.639)	0.002 **	2.981 (1.335–6.660)	0.008 **	2.429 (1.069–5.518)	0.034 *
**Step 2**						
**Ever received breast health education**						
No			1.000		1.000	
Yes			1.880 (1.104–3.201)	0.020 *	1.752 (1.026–2.992)	0.040 *
**Step 3**						
**Marital status**						
Single					1.000	
Married					1.267 (0.713–2.252)	0.420
**Location of residence**						
Rural areas					1.000	
Urban areas					1.961 (1.059–3.632)	0.032 *

SESI, socioeconomic status index; OR, odds ratio.

^a^ Crude OR

^b^ Adjusted for health education

^c^ Adjusted for breast health education, marital status, and location of residence.

Step 1 model fit, −2 log likelihood = 538.866, Cox and Snell R^2^ = 0.008, Nagelkerke R^2^ = 0.031, Model *χ*^2^ = 15.076 (*p* < 0.001).

Step 2 model fit, −2 Log likelihood = 533.210, Cox and Snell R^2 =^ 0.011; Nagelkerke R^2^ = 0.043, Model *χ*^2^ = 20.733 (*p* < 0.001).

Step 3 model fit, −2 log likelihood = 525.983, Cox and Snell R^2 =^ 0.015; Nagelkerke R^2^ = 0.057, Model *χ*^*2*^ = 27.960 (*p* < 0.001).

*P*-Values were calculated using logistic regression analysis.

** *p* < 0.01

* *p* < 0.05.

Model 1 (the crude model) showed that women in the medium to high SES index groups had significantly higher odds of BSE and CBE than those in the low SES index group. However, women in the medium SES index group were more likely to undergo mammography. Specifically, women with medium and high SES indexes had 2.113 (95% CI: 1.514, 2.948) and 3.323 (95% CI: 2.423, 4.556) times the odds of performing BSE than women with low SES indexes; 1.863 (95% CI: 1.245, 2.790) and 3.320 (95% CI: 2.285, 4.822) times the odds of undergoing CBE than women with low SES index; and 3.739 (95% CI: 1.690, 8.274) and 3.453 (95% CI: 1.561, 7.639) times the odds of screening mammography than women with low SES indexes.

Model 2 showed that the strength of the associations between the SES index and three breast cancer screening practices slightly decreased after the addition of the following item: “Ever received breast health education”. However, the odds trend was consistent with the results of regression model 1. Controlling for other variables, breast health education was positively linked to female screening utilization. Specifically, women who received breast health education had 3.017 (95% CI: 2.333, 3.902), 2.029 (95% CI: 1.514, 2.718), and 1.880 (95% CI: 1.104, 3.201) times the odds of engaging in BSE, CBE, and mammogram practice than women who did not receive breast health education.

Model 3 showed that the strength of the influence of the SES index on female breast cancer screening practices decreased after the addition of marital status and the location of residence based on model 2. However, the odds trend was still consistent with the results of model 1. Controlling for other variables, married urban women had higher odds of engaging in breast cancer screening than single rural women. Specifically, married women had 2.183 (95% CI: 1.630, 2.923) and 1.693 (95% CI: 1.212, 2.364) the odds of practicing BSE and CBE than unmarried women, and urban women had 1.484 (95% CI: 1.122, 1.963), 1.725 (95% CI: 1.238, 2.404), and 1.961 (95% CI: 1.059, 3.632) the odds of participating in BSE, CBE, and mammogram practice than rural women.

## Discussion

To the best of our knowledge, this study provides the first evidence of the relationships between female SES and breast cancer screening practices using the composite SES index. Consistent with findings of previous studies [19–23], our results confirmed that socioeconomic disparities exist in breast cancer screening utilization in Eastern China. SES indicators (education level, annual household income, and occupation) were positively associated with the utilization of breast cancer screening. In addition, we found that the composite SES index was an important factor related to female breast cancer screening practices. Our findings provide an evidence base for policy formulation to design intervention strategies and improve the screening uptake in China, and ultimately promote more equitable breast cancer outcomes.

Our results demonstrated that the rates of breast cancer screening practices were critically low among women living in Eastern China, which needs improvement. Most local women failed to participate in breast cancer screening. This implies that several complex factors may be at work. An important reason is that China still has no nationwide population-based breast cancer screening program because of the large population, insufficient screening equipment, funding obstacles, and inadequate insurance coverage [13]. Consequently, the government should put forth greater efforts to increase the coverage and penetration of breast cancer screening. Specifically, it is urgently needed to establish population-based national breast cancer screening programs and national mass-media campaigns to increase participation rates.

Our results showed that higher education, income, and occupation increased the likelihood of participation in breast cancer screening. Similar results are reported in other countries, and they show the positive impact of socioeconomic factors such as education [21, 23, 42, 43], income [21, 23, 42], and occupation [42, 43] on breast cancer screening. Among these SES components, education is considered a prerequisite for health, reflecting health awareness, and decision-making competence [26]. Occupation reflects prestige and skill-related achievements [26]. Income reflects the ability to pay for more health inputs, which is closely linked to the level of education and occupation [26, 27]. This implies that higher education, higher income, and belonging to higher occupational classes were related to healthier lifestyles, access to more health promotion resources, and easier access to preventive healthcare services [23]. Socioeconomic disparities in breast cancer screening participation provide valuable information for public health programs. Reducing breast cancer screening disparities requires policy initiatives to address SES component inequalities.

Another noteworthy finding of our study was that the composite SES index was an independent determinant of breast cancer screening practices. We found that women with higher SES indexes had a greater likelihood of breast cancer screening participation. Regression analysis also showed a strong influence of the SES index on breast cancer screening. This highlights the importance of SES in influencing female breast cancer screening in Eastern China. Our results are similar to the findings of other international studies [19–23, 44], reporting that lower SES was a major determinant for non-participation in breast cancer screening. It is imperative to develop optimal national breast screening programs to increase promotion of early screening among women with low SES and who are under-screened.

Notably, our results indicate that a higher SES index has a positive impact on women’s breast cancer knowledge. Similarly, a study by Yavari et al. [45] showed that women with lower education had insufficient knowledge of breast cancer detection, compared with highly educated women. This indicates that women with high SES tend to have more health-related knowledge [24], which may contribute to their participation in health-promoting programs [16]. Educational campaigns about cancer should proactively reach vulnerable women and promote widespread access to knowledge about breast cancer.

Our study also found that the influence of the SES index on breast cancer screening practices varies with health education, marital status, and location of residence. This demonstrates an association between the SES index and these characteristics of women. In addition, being married, receiving breast health education, and living in urban areas increased the odds of breast cancer screening practices. Rakowski et al. [46] in the United States and Al Rifai et al. [23] in Jordan highlighted the positive impact of health education on participation in breast cancer screening. Furthermore, results from a systematic review documented that being single and living in a rural area were associated with the low participation in breast cancer screening in Latin America [21]. Therefore, cancer screening interventions need to take these factors into account.

It is worth noting that we found that the SES index is a neutral and stable variable, which did not alter the relationship of three SES indicators with breast cancer screening in the study population. Previous studies analyzed separate bivariate associations between selected SES indicators (income or education) and breast cancer screening [19–21, 23, 42, 43]. However, each dimension of SES can only reflect different actual resources. The SES index is represented as a new comprehensive indicator [27, 40]. Compared with a single indicator approach, the SES index can be used to explore the composite contributions of educational attainment, income, and occupation to breast cancer screening uptake. Similarly, scholars in other fields also confirmed that the SES index is a robust measure for SES assessment [27, 39, 40].

Several potential limitations should be considered while interpreting our results. The main challenge with cross-sectional design is the inability to provide inferential causality. Potential sampling bias should be noted because people willing to participate were enrolled in this study, that is, the sample was not randomly obtained from the source population. Some limitations of self-report questionnaires, including item clarity, social desirability, or response bias, may have also affected the reliability of the study results. This survey was conducted during the COVID-19 pandemic, and thus, breast cancer screening practices during this time may not reflect true rates. Of note, other useful information was not included in this study, such as health insurance [21, 22], health status [20, 42], and unhealthy behaviors [23, 42]. Furthermore, SES is constantly changing throughout one’s life, which may have influenced our findings. Also, there is no indication of whether the current SES index (as measured) is related to screening behaviors which may have occurred many years in the past. Finally, according to the CACA guidelines at the time, BSE and CBE should begin at the age of 20, and mammography screening should begin at 40 years. We included women 19–39 years of age in our analysis for both BSE, CBE, and mammography screening utilization. Not aligning our study design precisely with recommendations provided by the CACA poses as a limitation in our study design. Notwithstanding the limitations described, our study provides useful information for designing breast cancer screening intervention strategies and promoting more equitable breast cancer outcomes. Our findings could be used as a historical reference. In the future, more in-depth, well-designed longitudinal and prospective research will be required to address these potential study limitations.

## Conclusions

This study found that female breast cancer screening rates were low among Eastern Chinese women, despite its increasing prevalence. Socioeconomic inequalities in the patronage of female breast cancer screening exist in Eastern China. Furthermore, socioeconomic factors strongly influence breast cancer screening practices and breast cancer-related knowledge. Most importantly, using the composite SES index, this study confirmed the effect of SES on female breast cancer screening practices. The composite SES index was found to be positively and significantly correlated with female breast cancer screening patronage. There was an interaction between the SES index and those characteristics, such as health education, marital status, and location of residence. Our results provide strong evidence further validating that SES is a multidimensional construct, and the SES index is a robust variable for SES assessment. Future breast health promotion programs should develop tailored interventions targeting these women with lower SES to promote the patronage of breast cancer screening, especially lower educational levels, insufficient income, and lack of employment.

## Supporting information

S1 File RAW data. (XLSX)

S2 File RAW data SPSS. (SAV)
